# The Essential Elements of Ideal Surgery

**Published:** 1894-03

**Authors:** B. F. Wilson

**Affiliations:** Slater, Missouri; Surgeon Chicago and Alton Railroad, etc.


### THE ESSENTIAL ELEMENTS OF IDEAL SURGERY.*

By B. F. WILSON, A. M., M. D.

Slater, Missouri.

Surgeon Chicago and Alton Railroad, etc.

In surgery the ideal is realized in any given case when the results show that conservatism and utility inspired the conception. To retain a part rendered useless by loss of counterparts, or to save all tissue possible in every case, would only serve to maim and burden the patient—a triumph of conservatism so *ultra* that instead of receiving plaudits, it should be classed as malpractice.

The relative powers of regeneration of the various tissues should receive thoughtful consideration by every operator in reaching his conclusions. The osseous tissues of the leg may be literally comminuted, yet if the vascular tissues be sufficiently intact, amputation should be done only as a *dernier ressort.* On the other hand, the soft and vascular parts of the leg may be so mutilated and torn with little or no injury to the bone that amputation becomes not only conservative but imperative.

*Read before the Pan American Medical Congress.

Railroad surgery, while comprising all that is common to general surgery, embraces much more in the superadded elements of crushing force and violence, impressing features distinctly peculiar to railroad traumatisms. Thus, while the principles of general surgery must ever form the *substrata* on which to predicate a practice, yet a due recognition of the overwhelming destructiveness of railroad injuries must obtain and govern the operator, else dire and disappointing results legitimately follow. Two injuries, alike in all essential respects as to parts involved and extent of injury, yield widely different results, even if treated by the same surgeon; in one case the injury is local, the tissues as a whole are not involved, a bone is broken or an artery severed, with little or no destruction of contiguous tissue, entailing upon the economy but a slight wave of nervous sympathy; whereas, in the other injury, caused by a ponderous body impelled with great *momentum*, the bone is so crushed and disintegrated as virtually to constitute a vital amputation, the physical amputation being merely perfunctory.

The central idea, the one guiding thought to attain the ideal in railroad surgery, is ever to keep in mind the radical difference between railroad traumatisms and ordinary injuries, the cause of this difference in gravity, to wit: the crushing violence in railroad injuries that involve not only the physical integrity of the parts, but also overwhelm the vitality in nervous shock and physical collapse. Experience teaches the railroad surgeon that a departure from the rules of procedure obtaining in general surgery, as in amputation, etc., is essential to success; that in civil practice the ideal operation is to attempt to save all tissue compatible with utility, and that an immediate operation is to be preferred, the primary the exception. Just the reverse should be the rule in railroad injuries to secure good results, the conservation of tissue being of minor consideration. First, and above all else, give time for reaction to fairly set in that the involvement of the vascular system may be accurately determined, and serve as a guide as to the extent of tissue to be sacrificed. There is abundant cause why reaction should be well established in railroad injuries before an operation is done. In the crushing and mangling not only are the nerves subject to such destructive violence as to destroy their function, but likewise the vascular system suffers from the same cause, hence the absolute necessity of awaiting reaction in all major cases, as loss of nervous innervation in any part will determine to a great extent the site of election. The regeneration of nervous tissue will be too tardy to save the soft parts when the integrity of the nervous system has been seriously impaired, hence the wisdom of the rule to cut wide of the injury in immediate operations in railroad traumatisms, is confirmed by both observation and experience, and, although the soft parts *seem* to be *intact*, yet the uniform sloughing attendant upon immediate operations teaches us to estimate duly the wide difference in the *causation* of injuries, one local *per se*, the other involving, in the violent stretching and tearing of the soft parts, the whole nervous system.

Unlike bone and nerve tissue, the blood vessels, when their continuity is destroyed, cannot be reproduced or their patency restored; hence, the entire dependence of all other parts of the economy upon the unbroken continuity and functional integrity of the blood vessels, for while compensatory collateral circulation may in time be established, yet this wondrous provision of nature may prove wholly inadequate to supply in sufficient amount the blood pabulum so essential to the life of the part. To this inherent, tardy delay in the development of the collateral circulation, the loss of many parts is due, even when concomitant injuries are of a trivial nature.

Looking to attainable ideal results, it is well to consider the relative vital powers of resistance of the various tissues in order to fully estimate the loss of nervous innervation when the vascular supply is inadequate, due to their common impairment.

In many cases the nerve lesion is disproportionately greater than the vascular, and thus fail of rapid reparation for want of nervous innervation. In some injuries, due to wrenching, stretching and tearing, the tortuosity of the arteries leaves them comparatively intact, whilst the nerves suffer such stretching and physical injury as to sever the continuity of their axis cylinders, resulting in abolition of function more or less enduring. As effect follows cause, so may we ascribe the greater shock and collapse as distinctive and peculiar to railroad injuries; not sufficient blood has been lost nor destruction of tissue to thus overwhelm and imperil life, but in the general injury to the nervous system we may find abundant cause.

*Dressings*.—Means should be adapted to ends. Hemostasis should be effected by torsion, as this method greatly lessens the labor of the surgeon in the future management of the case and exempts the patient from complications due to other methods. In determining the choice of local applications to be used in any given case, a thoughtful discrimination is demanded. As in medicine, so in surgery, each particular case is a law unto itself. In each individual case the surgeon is confronted with conditions ranging from a simple aseptic incised wound to one all "tattered and torn," beset with *auto*- and *exo*-septic *materies morbi*. The conditions are so diverse that manifestly the treatment so imperatively demanded in the one case would only delay or prevent restoration in the other.

The disinfection of pathogenic tissues is the fulfillment of ideal surgery as to dressings—a conception of science, upheld by incontrovertible facts, and when an infected wound is once rendered aseptic by the use of disinfectants, their further use is not only contraindicated, but detrimental in the extreme by inviting destructive and devitalizing stimulation, in many cases eventuating in necrosis instead of normal repair.

